# Can silicon carbide serve as a saturable absorber for passive mode-locked fiber lasers?

**DOI:** 10.1038/srep16463

**Published:** 2015-11-12

**Authors:** Chih-Hsien Cheng, Yung-Hsiang Lin, Ting-Hui Chen, Hsiang-Yu Chen, Yu-Chieh Chi, Chao-Kuei Leeb, Chih-I Wua, Gong-Ru Lin

**Affiliations:** 1 Graduate Institute of Photonics and Optoelectronics, Department of Electrical Engineering, National Taiwan University (NTU), No. 1, Sec. 4, Roosevelt Road, Taipei 106, Taiwan R.O.C; 2 Department of Photonics, National Sun Yat-sen University, No. 70, Lien-Hai Rd, Kaohsiung 804, Taiwan

## Abstract

The study presents a novel demonstration of a passively mode-locked erbium-doped fiber laser (EDFL) that is based on a silicon carbide (Si_x_C_1−x_) saturable absorber. When the C/Si composition ratio is increased to 1.83, the Si_x_C_1−x_ film transforms from two-photon absorption to nonlinear saturable absorption, and the corresponding value reaches −3.9 × 10^−6^ cm/W. The Si-rich Si_x_C_1−x_ film cannot mode lock the EDFL because it induced high intracavity loss through two-photon absorption. Even when a stoichiometric SiC is used, the EDFL is mode locked, similar to an EDFL operating under weak nonlinear-polarization-rotation condition. A C-rich Si_x_C_1−x_ film containing sp^2^-orbital C–C bonds with a linear absorbance of 0.172 and nonlinear absorbance of 0.04 at a 181 MW/cm^2^ saturation intensity demonstrates nonlinear transmittance. The C-rich Si_x_C_1−x_ saturable absorber successfully generates a short mode-locked EDFL pulse of 470 fs. The fluctuation of the pulse-train envelope dropps considerably from 11.6% to 0.8% when a strong saturable-absorption-induced self-amplitude modulation process occurs in the C-rich Si_x_C_1−x_ film.

Mode-locked fiber lasers have been widely used for biophotonic imaging^1^, micromachining^2^, and microwave generation^3^, which can typically be conducted through intensity/phase modulation^4^,^5^, nonlinear Kerr lensing^6^, and saturable absorption^7^,^8^,^9^,^10^. Saturable absorbers are typically used to initiate passive mode-locking in lasers. As early as 1996, Kelly and coworkers employed semiconductor saturable absorber mirrors (SESAMs) in pulsating solid-state lasers^11^. Using a specific SESAM, Jung *et al.* further improved the mode-locked laser pulsewidth to a sub-10-fs regime^12^. However, SESAMs typically employed in solid-state lasers can cause higher insertion loss because of the absence of focusing geometry when reflection-type SESAMs are positioned in the linear or ring fiber cavity. Therefore, transmission-type saturable absorbers are used for passive mode locking in all fiber laser systems since the aforementioned study of Jung *et al.* In 2004, Set and coworkers reported a demonstration of a single-walled carbon nanotube (CNT)-based saturable absorber applied for subjecting an erbium-doped fiber laser (EDFL) to passively mode lock^13^. The bandgap of a CNT can be detuned by changing its diameter and number of walls to induce saturable absorption for mode locking at different wavelengths^14^,^15^. After the development of the CNT saturable absorber, several types of carbon based materials including the graphene^16^,^17^,^18^,^19^, the graphite^20^,^21^, the graphene oxide^22^,^23^, and the charcoal powder^24^,^25^, were successively introduced for use as saturable absorbers for subjecting EDFLs to passively mode lock^24^,^25^. More recently, even topological insulator materials have been considered for use as saturable absorbers^26^,^27^. A disadvantage of using graphene and other carbon-based materials is that they may be damaged at a high intracavity laser intensity, resulting in the inevitable degradation of the mode-locking performance at a damage threshold intensity; the damage threshold intensity is 0.28 TW/cm^2^ for graphene^28^. Raising the damage threshold of saturable absorbers is necessary for pulsating high-power mode-locked EDFLs.

Because of its high thermal stability and high chemical inertness^29^, nonstoichiometric silicon carbide (Si_x_C_1−x_) is a candidate for a high-damage-threshold material, with a damage threshold that can reach up to 40 TW/cm^2^^30^. This material has already been considered for high-temperature and high-power electronics and has a wide variety of optoelectronic applications^31^,^32^. In particular, Si_x_C_1−x_ is a potential saturable absorber for passively mode-locked fiber lasers because of its aforementioned excellent features^30^. Furthermore, its optical nonlinearity has been investigated in recent years. DesAutels *et al.* studied the damage threshold and optical nonlinearity of bulk Si_x_C_1−x_, determining that the nonlinear absorption coefficient and refractive index of the semiinsulating Si_x_C_1−x_ were 0.064 cm/GW and 4.75 × 10^−6^ cm^2^/GW, respectively^30^. Ding and coworkers observed the third-order nonlinear optical susceptibility of nitrogen-doped Si_x_C_1−x_ at various doping concentrations^33^. The real parts of the third-order nonlinear susceptibilities could be increased up to 6.16 × 10^−13^ esu by increasing the doping concentration to 2 × 10^17^ cm^−3^ 33. In addition, the carbon content of an Si_x_C_1−x_ film could be detuned to increase sp^2^-orbital C–C bonds, which enhanced the ultrafast optical nonlinearity.

The current study presents a novel demonstration of the use of a nonstoichiometric Si_x_C_1−x_ film as a saturable absorber for passively mode-locked fiber lasers. Nanoscale Si_x_C_1−x_ films with different C/Si composition ratios were grown using a hydrogen-free plasma-enhanced chemical vapor deposition (PECVD) system at relatively low substrate temperatures. The optical nonlinearity of the nanoscale Si_x_C_1−x_ films was observed by varying the stoichiometry of the Si_x_C_1−x_ film from Si-rich to C-rich conditions. The effect of sp^2^-orbital C–C bonds on the nonlinear transmittance of Si_x_C_1−x_ was characterized to determine methods through which the phase-focusing performance of mode-locked EDFLs can be improved according to C-rich Si_x_C_1−x_ films.

## Results

### Material and optical analyses of Si_x_C_1−x_ saturable absorbers

**Figure 1:**
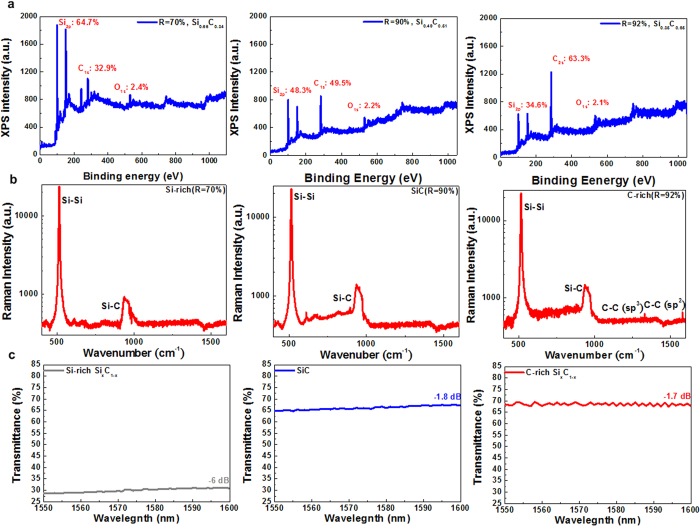
Material characteristics of Si_x_C_1−x_ films. (**a**) The XPS spectra and (**b**) Raman scattering spectra of the Si-rich (left), stoichiometric (middle), and C-rich (right) Si_x_C_1−x_ films. (**c**) The linear transmittance of the Si_x_C_1−x_ films.

Figure 1a shows X-ray photoelectron spectroscopy (XPS) spectra of the Si_x_C_1−x_ films with dissimilar C/Si composition ratios. The C/Si composition ratio of the Si_x_C_1−x_ films increases from 0.51 to 1.83 when the [CH_4_]/[CH_4_ + SiH_4_] fluence ratio is increased from 70% to 92%, which corresponds to a decrease in the films’ fraction index *x* from 0.66 to 0.33. Specifically, the O/Si composition ratio decreases to 0.06 when the [CH_4_]/[CH_4_ + SiH_4_] fluence ratio is increased to 92%, which enhances the quality of the PECVD-grown Si_x_C_1−x_ film without oxidation. A higher quantity of Si-C or C-C bonds can be formed at a higher [CH_4_]/[CH_4_ + SiH_4_] fluence ratio because a higher quantity of decomposed carbon atoms can be deposited to the Si_x_C_1−x_ films. In general, decomposing SiH_4_ molecules is easier than decomposing CH_4_ molecules because the dissociation energy of SiH_4_ molecules is lower (75.6 kcal/mol)^34^.

At a low temperature and in a weak RF plasma environment, each molecule of a reactant with a high molecular density gains insufficient energy from the RF plasma, resulting in the decomposition rates of SiH_4_ and CH_4_ being similar. In particular, the average dissociation energy of a reactant molecule decreases when the total reactant fluence is increased by increasing the [CH_4_]/[CH_4_ + SiH_4_] fluence ratios, which further degrades the decomposition rate of the SiH_4_ molecules. Therefore, a Si_x_C_1−x_ film grown at a higher [CH_4_]/[CH_4_ + SiH_4_] fluence ratio exhibits a lower quantity of excessive Si atoms, which corresponds to a higher C/Si composition ratio. To determine the bonding structures of the Si_x_C_1−x_ films, a Raman scattering analysis is applied to the Si_x_C_1−x_ films involving different C/Si composition ratios (Fig. 1b). The Raman scattering spectra reveal relatively strong scattering peaks at 970 cm^−1^, which can be attributed to the longitudinal optical (LO) modes of the Si-C bonds^35^. Furthermore, the Si-related Raman scattering peak at 520 cm^−1^ monotonically decreases as the C/Si composition ratio increases from 0.51 to 1.83. This is because more carbon atoms are incorporated into the Si_x_C_1−x_ film during the growing process under CH_4_-rich conditions. As evidence, the C-rich Si_x_C_1−x_ film reveals additional Raman scattering peaks at 1330 and 1580 cm^−1^, and these peaks can be attributed to the sp^3^- and sp^2^-orbital C-C bonds, respectively^36^,^37^. The quantity of C-C bonds in the C-rich Si_x_C_1−x_ film is too low to be observed in the Raman scattering signals.

**Figure 2:**
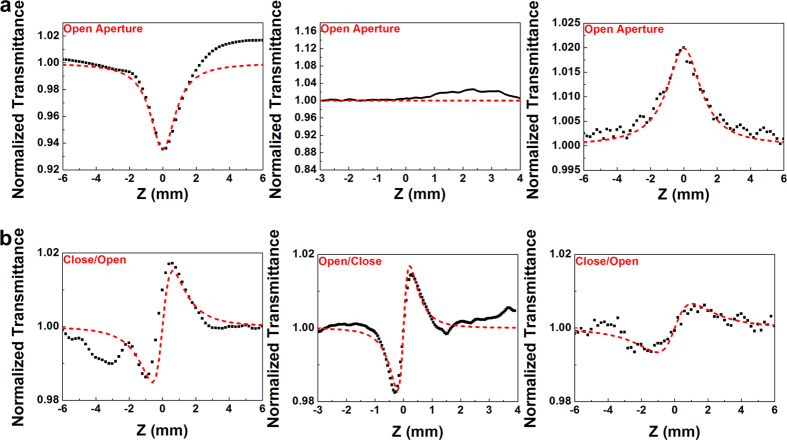
Z-scan traces of Si_x_C_1−x_ films. (**a**) Open-aperture Z-scan traces and (**b**) closed-aperture/open-aperture Z-scan curves of the Si-rich (left), stoichoimetric (middle), and C-rich (right) Si_x_C_1−x_ films.

Figure 1c illustrates a linear transmittance spectrum of the Si_x_C_1−x_ films with various C/Si composition ratios. When the Si_x_C_1−x_ film is transformed from a C-rich to a Si-rich condition, the transmittance of the film decreases considerably from 0.68 to 0.3. The Si-rich Si_x_C_1−x_ film demonstrates a smaller bandgap and higher absorbance at longer wavelengths, which inevitably causes higher loss. The higher loss can increase the EDFL lasing and mode-locking thresholds. To further prove the optical nonlinearity of the Si_x_C_1−x_ films involving various C/Si composition ratios, their closed- and open-aperture Z-scan traces are examined (Fig. 2a). The normalized transmittance of the open-aperture Z-scan trace of the Si-rich Si_x_C_1−x_ film gradually decreases as the peak intensity of the pump beam increases, indicating that a strong two-photon absorption phenomenon occurs in the Si-rich Si_x_C_1−x_ films and that the phenomenon weakens in C-rich Si_x_C_1−x_ films with higher C/Si composition ratios. In general, the two-photon absorption coefficient can be obtained from the Z-scan analysis by using the following formula^38^:


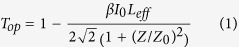


where *β* denotes the nonlinear absorption coefficient of the Si_x_C_1−x_ film, *I*_*0*_ is the peak intensity of the pump beam, *L*_eff_ is the effective thickness of the Si_x_C_1−x_ film, and *Z*_*0*_ is the Rayleigh length of the pump beam.

After fitting, the two-photon absorption coefficient of the Si-rich Si_x_C_1−x_ film is determined as 4.6 × 10^−6^ cm/W. The two-photon absorption phenomenon occurrs mainly because the production of excessive Si atoms reduces the bandgap of the Si-rich Si_x_C_1−x_ film. By contrast, a stoichiometric Si_x_C_1−x_ film exhibits a larger bandgap and results in a lower two-photon absorption coefficient. In particular, neither two-photon absorption nor saturable absorption is observed in the stoichiometric Si_x_C_1−x_ film in the open-aperture Z-scan analysis. When the carbon content of a Si_x_C_1−x_ film is increased to attain C-rich conditions, the intensity-dependent absorption in the C-rich Si_x_C_1−x_ film abruptly changes from two-photon absorption to saturable absorption because of the excessive amount of C atoms present in the C-rich Si_x_C_1−x_ film. In general, most carbon based materials exhibit saturable absorption. Embedding either diamond-like or graphite-like carbon particles in the C-rich Si_x_C_1−x_ film may improve the saturable absorption effect in the film; however, the sp^2^-orbital C-C bonding structure, rather than the sp^3^-orbital C–C bonds, may dominate the saturation process. The saturable absorption coefficient of the C-rich Si_x_C_1−x_ film is derived as −3.9 × 10^−6^ cm/W by fitting the open-aperture Z-scan curve of the film with Eq. (1).

To exclude the intensity dependent absorption, closed-aperture/open-aperture Z-scan traces are obtained by dividing the closed-aperture Z-scan trace with the open-aperture Z-scan trace (Fig. 2b). Furthermore, the nonlinear refractive index can be obtained by fitting a theoretical transmittance function to the Z-scan analysis data as follows^38^:


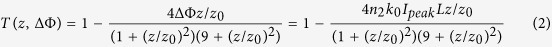


where ΔΦ, *n*_*2*_, and *k*_*0*_ denote the phase shift, nonlinear refractive index of the Si_x_C_1−x_ film, and wave number, respectively. A numerical simulation reveals that the nonlinear refractive index of the Si_x_C_1−x_ film varying from 1.83 × 10^−11^ to 3.86 × 10^−11^ cm^2^/W when the C/Si composition ratio is increased from 0.51 to 1.83. This is because the excessive C content demonstrates a more effective contribution to the high nonlinear optical properties of the Si_x_C_1−x_ film. In principle, the nonlinear refractive index, which is defined as a function of the third-order nonlinear susceptibility of centrosymmetric materials, can be enhanced by reducing the lattice constant, effective mass, or both parameters, as shown in the following equation^39^:


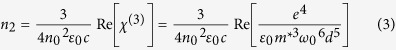


where *χ*^*(3)*^, m*, *n*_*0*_, and *d* denote the third-order nonlinear susceptibility, effective mass, linear refractive index, and lattice constant of the centrosymmetric material, respectively. The variables *e*, *c*, *ε*_*0*_, and *w*_*0*_ represent the electric charge, light velocity in the vacuum, vacuum permeability, and resonant frequency, respectively.

In the Si-rich Si_x_C_1−x_ film, excessive Si atoms replace C atoms to form a higher quantity of Si-Si bonds in the Si_x_C_1−x_ matrix. Such Si-Si bonds increase the lattice constant (5.431 Å) and effective mass (0.36*m*_0_), which can seriously degrade the third-order nonlinear susceptibility. The Si network typically possesses a higher linear refractive index but provides a lower nonlinear refractive index in the Si-rich Si_x_C_1−x_ film. Conversely, the C-C bonds in C-rich Si_x_C_1−x_ reduce the effective lattice constant and effective mass considerably, increasing the nonlinear refractive index. As expected, the Si_x_C_1−x_ films containing a higher quantity of sp^3^-orbital C-C bonds show a higher lattice constant (4.5396 Å) and a higher effective mass (0.57*m*_0_) than those with only Si-C bonds. Moreover, a higher quantity of sp^3^-orbital C-C bonds in the C-rich Si_x_C_1−x_ films result in a lower C-rich Si_x_C_1−x_ nonlinear refractive index. By contrast, the sp^2^-orbital C-C bonds considerably reduce the lattice constant (2.46 Å) and effective mass (0.04 *m*_0_ to 0.06 *m*_0_) of the C-rich Si_x_C_1−x_ films, effectively increasing the nonlinear refractive index of the C-rich Si_x_C_1−x_ film. If the sp^2^-orbital C-C bonds are transformed to graphene-like C-C bonds, the effective carrier mass of the matrix can be reduced to zero, thereby increasing the nonlinear refractive index to the maximal value. In the present study, the sp^2^-orbital C-C bonds dominate the nonlinear refractive index of the C-rich Si_x_C_1−x_ films. This is confirmed by the Raman scattering analysis, thus clearly explaining the reason for the C-rich Si_x_C_1−x_ film with enriched graphite-like C-C bonds demonstrating a higher nonlinear refractive index than those of the Si-rich and stoichiometric Si_x_C_1−x_ films.

### Unstable passive mode locking of EDFL without a saturable absorber

**Figure 3:**
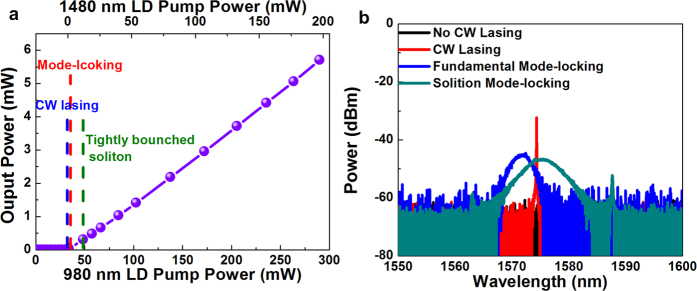
Optical properties of the EDFL without a saturable absorber. (**a**) *P*–*I* curve and (**b**) optical spectra (under various mode-locking conditions) of the passively mode-locked EDFL without a saturable absorber.

Figure 3a depicts plots of the output power versus the forward (980 nm) and backward (1480 nm) pumping powers for an EDFL with an unstable passive mode locking and without a saturable absorber. When the bidirectional 980-nm/1480-nm LD pumping power is set to 30 mW/0.1 mW, the EDFL reaches the continuous-wave (CW) lasing threshold for the narrow-linewidth spectrum (Fig. 3b). The EDFL switches its lasing state from CW to the fundamental mode-locking state when the forward/backward pumping power is slightly increased to 33 mW/0.72 mW. Although the mode locking of an EDFL without a saturable absorber can be initiated through a loss modulation induced by the residual nonlinear polarization rotation (NPR) effect, the permissible detuning of the polarization controller tilting angle is strictly limited to a narrow range because intracavity polarization changes can easily cause the effect gain to drop to a level lower than the mode-locking threshold. When the forward/backward pumping power is increased to 48 mW/7.84 mW, the high-power soliton pulse train of the fundamentally mode-locked EDFL begins to split into tightly bunched soliton pulses because high-order solitons are not permitted in the EDFL intracavity. When the EDFL is operated at a level lower than the 30 mW/0.1 mW of the forward/backward pumping power, the CW lasing optical spectrum having a narrow peak at 1575 nm cannot be observed because the pumping gain do not compensate for the EDFL intracavity loss (Fig. 3b). The optical spectrum is gradually broadened by increasing the pumping power to 33 mW/0.72 mW for achieving the mode-locking threshold, and the fundamentally mode-locked pulse is progressively transformed to fundamental soliton pulses by further increasing the pumping gain. In addition, the considerably broadened optical spectrum is accompanied by an appreciable Kelly sideband at slightly red-shifted wavelengths.

**Figure 4:**
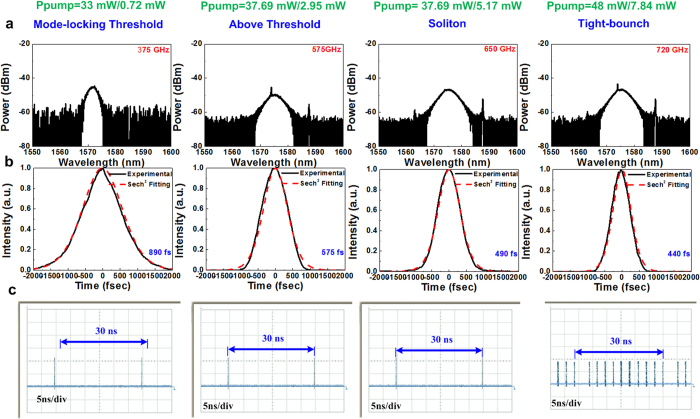
Performance of the EDFL without a saturable absorber at dissimilar pumping operations. (**a**) Optical spectra, (**b**) temporal pulse, and (**c**) pulse train of the passively mode-locked EDFL without a saturable absorber at diverse pumping operations.

Figure 4a shows the evolution of the spectrum, waveform, and trace of the pulse of a passively mode-locked EDFL without a saturable absorber when the pumping power is gradually increased. The fundamentally mode-locked EDFL pulse is relatively unstable and fluctuated; it dropped appreciably from 890 to 440 fs when the spectral linewidth is broadened from 3.25 to 5.98 nm by inreasing the forward/backward pumping power from 33 mW/0.72 mW to 48 mW/7.84 mW (Fig. 4b). The time-bandwidth product (TBP) of the mode-locked pulse varies from 0.334 to 0.317 (close to the transform limit). Nevertheless, the pulse shortening resulting from the enhancement of the peak power of the circulated pulse is intrinsically restricted to increase the soliton order. Consequently, the increased pumping power prompts only the gain of the fundamentally mode-locked pulse in the EDFL cavity, which gradually enhances the self-amplitude modulation (SAM) depth to further reduce the pulsewidth and amplify the peak power to its upper limit. Nevertheless, the increase in the gain cannot infinitely amplify the peak power of the pulse because the soliton order is limited after entry into the fundamental soliton regime. Figure 4c shows that the sampled traces of a pulse train and the pulse timing of the passively mode-locked EDFL become unstable with an increase in the jitter and split bunch soliton pulse within one period because of the aforementioned limitation. Normally, the pulse train obtained from the EDFL at the mode-locking threshold (at a forward/backward pumping power of 30 mW/0.1 mW) shows a clear trace with a repetition rate of 33 MHz. Increasing the pumping power to 48 mW/7.8 mW provides a fundamentally mode-locked pulse train with a relatively unstable but observable trace. At a pumping power greater than that required for generating the fundamental soliton, the single pulse is moderately blurred by a tightly bounded soliton bunch with a single period because the extremely high intracavity gain causes the intense fundamental soliton to be split into multiple solitons.

**Figure 5:**
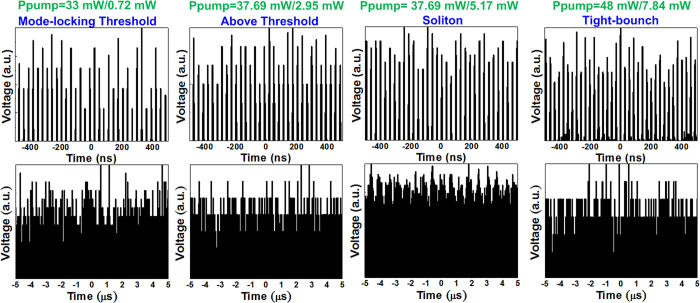
Stability of the EDFL without a saturable absorber at different pumping operations. Stability of the passively mode-locked EDFL without a saturable absorber on different time scales.

To characterize, the stability of the beam intensity of the passively mode-locked EDFL without a saturable absorber is calculated for different time scales (Fig. 5). In principle, the carrier amplitude jitter (CAJ) is used to determine the pulse quality in the time domain. The CAJ can be defined as CAJ (%) = (*σ*/*I*_avg_) × 100, where *σ* and *I*_avg_ denote the standard deviation of the peak pulse intensity and the average pulse intensity, respectively. The CAJ of the passively mode-locked EDFL improves from 11.2% to 5.12% when the pulse generation varies from the fundamental mode-locking condition to the soliton condition. The improvement results from insufficient SAM in the absence of a saturable absorber; SAM synchronizes only the phase of a few longitudinal modes for a finite modulation depth. An increase in either the phase or amplitude jitter degrades the performance of the passively mode-locked EDFL. Increasing the pumping gain may synchronize the relative phase of additional longitudinal modes to improve the pulse stability. The CAJ of the EDFL is degraded to 6.53% with an increase in the pumping power because the single soliton pulse is split into tightly bound soliton bunch pulses within one period. For an extremely high intracavity gain, both the amplitude jitter and phase jitter are degraded because the split multiple solitons are randomly located without constant spacing in the time domain.

### Stabilized passive mode locking of EDFL with a Si_x_C_1−x_ saturable absorber

**Figure 6:**
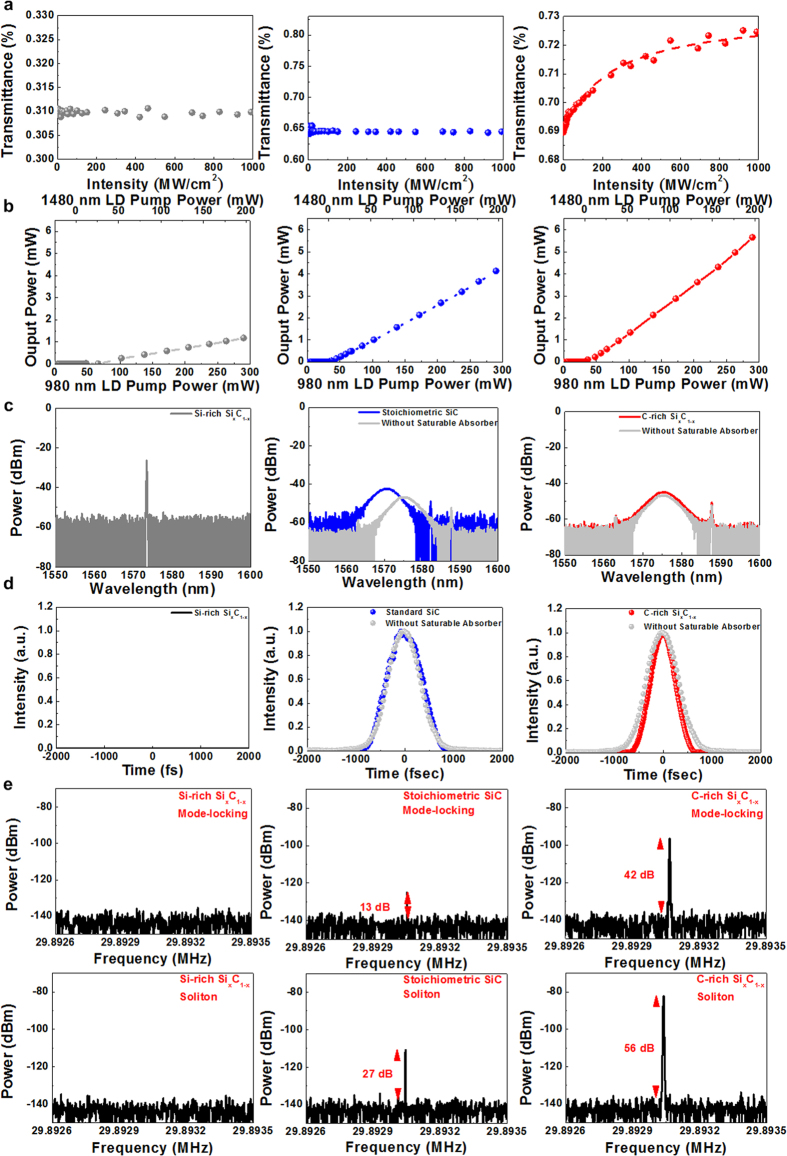
Performance of the EDFL with a Si_x_C_1−x_ saturable absorber. (**a**) Nonlinear transmittance, (**b**) *P*–*I* curve, (**c**) optical spectra, (**d**) temporal shape, and (**e**) RF signals of the fundamental mode-locking (Upper) and the solution (Lower) pulses of the passively mode-locked EDFL with a Si-rich (left), stoichiometric (middle), and C-rich (right) Si_x_C_1−x_ saturable absorber.

To achieve a stable mode locking, another SAM mechanism, such as saturable absorption, that is more robust than the NPR effect must be incorporated into the EDFL cavity. To confirm the functionality, nonlinear transmissions of Si_x_C_1−x_ saturable absorbers with different C/Si composition ratios are measured at various pumping powers of a pulsed laser (Fig. 6a). A saturable absorbance phenomenon is not observed for the Si-rich and stoichiometric Si_x_C_1−x_ films, which have the same thickness; however, the C-rich Si_x_C_1−x_ film exhibits a saturable absorbance when its nonlinear transform curve is fitted with the function *T* = exp{[−0.04/[1 + (*I*_in_/181 × 10^6^)]] −0.172}, where *I*_*in*_ is the pumping intensity. The observed linear and nonlinear absorbances are 0.172 and 0.04 , respectively, and the saturation intensity of the C-rich Si_x_C_1−x_ saturable absorber is 181 MW/cm^2^ (Fig. 6a). The high saturable absorbance is mainly attributed to the sp^2^-orbital C-C bonds in the film. The saturation intensity of the C-rich Si_x_C_1−x_ is considerably higher than that of graphene (1 MW/cm^2^) because the C-rich Si_x_C_1−x_ material demonstrates a lower quantity of sp^2^-orbital C-C bonds for saturable absorption. Therefore, the passive mode locking of an EDFL with a C-rich Si_x_C_1−x_ film is initiated at a higher and the EDFL exhibits a higher intracavity intensity compared with other lasers.

Furthermore, Fig. 6b shows *P*–*I* curves of EDFLs with Si_x_C_1−x_ saturable absorbers of different C/Si composition ratios. The slope d*P*/d*I* clearly increases from 1.4 × 10^−3^ to 6.85 × 10^−3^ W/A when the Si_x_C_1−x_ saturable absorber is transformed from an Si-rich absorber to a C-rich absorber, indicating that the total loss in the EDFL intracavity decreases when the linear absorption is reduced by changing the C/Si composition ratio. Therefore, the forward/backward pumping threshold of the EDFL drops from 53/8.45 to 35/1.62 when the Si_x_C_1−x_ saturable absorber is transformed from Si-rich to C-rich conditions; the forward/backward pumping threshold of 35/1.62 is higher than that of the EDFL without a saturable absorber because the Si_x_C_1−x_ saturable absorber can cause additional losses. When the Si_x_C_1−x_ film transfers from Si-rich to C-rich condition, the sample is still healthy under a pumping intensity of 0.8 GW/cm^2^. When increasing the pumping intensity to 1.27 GW/cm^2^ during the analysis of nonlinear transmittance, this sample is also not broken. Even illuminating by the femtosecond Ti:sapphire laser with a peak intensity of 10.46 GW/cm^2^, the sample is still not broken. In view of previous works, the pure Si and graphite have the lower damaged threshold intensities of 0.19 TW/cm^2^ and 1.44 TW/cm^2^, respectively, in comparison with that of 40 TW/cm^2^ for the SiC^30^,^40^,^41^. Therefore, the damaged threshold of Si_x_C_1−x_ film can be greatly increased as compared to other saturable absorbers. The Si_x_C_1−x_ has suffered from an optical intensity that is well below those damage thresholds mentioned in references.

To compare the performance of the passively mode-locked EDFL without a saturable absorber with that of a passively mode-locked EDFL with a saturable absorber, the passively mode-locked EDFL with an Si_x_C_1−x_ saturable absorber is operated with an effective gain identical to that of the passively mode-locked EDFL without a saturable absorber. The effective gain is set by precisely adjusting the forward/backward pumping power. For the fixed effective gain, the mode-locking mechanism of the EDFL containing a Si_x_C_1−x_ saturable absorber is concurrently determined by the weak NPR and the strong saturable-absorption-induced SAM. Figures 6c,d illustrate the optical spectra and autocorrelation traces of EDFLs passively mode locked by Si_x_C_1−x_ saturable absorbers with various C/Si composition ratios. For the Si-rich Si_x_C_1−x_ saturable absorber, the passively mode-locked EDFL cannot be operated at the same effective gain because of its lower nonlinear saturable absorption compared with the other saturable absorbers. When the stoichiometric Si_x_C_1−x_ film is used as the saturable absorber, the autocorrelated pulsewidth and spectral linewidth of the passively mode-locked EDFL are 492 fs and 5.02 nm, respectively, corresponding to a TBP of 0.321.

In comparison with the passively mode-locked EDFL without a saturable absorber, the EDFL containing the stoichiometric Si_x_C_1−x_ demonstrates a similar output because it also exhibits trivial saturable absorption, which is confirmed by its nonlinear transmittance. When the stoichiometric Si_x_C_1−x_ is used as the saturable absorber, the optical spectrum of the EDFL can be shifted because the NPR effect can contribute to the phase change during the polarization detuning process. This phenomenon induces the shift of the optical spectrum. Therefore, the spectrum peak differs from that of the EDFL without a saturable absorber. The mode-locking mechanism of the EDFL is attributed to the weak-NPR-induced SAM effect. By contrast, the C-rich Si_x_C_1−x_ film reduces the pulsewidth appreciably to 470 fs; the corresponding spectral linewidth is 5.13 nm, indicating a TBP of 0.319 (Fig. 6c,d, right column). For the same effective gain, the pulsewidth of the passively mode-locked EDFL involving a C-rich Si_x_C_1−x_ saturable absorber is shorter than that of the passively mode-locked EDFL without a saturable absorber. This is because the buried sp^2^-orbital C-C bonds enhance the saturable absorption, which then dominate the SAM process in the EDFL mode locking.

Moreover, the Fig. 6e shows the RF spectra of fundamental mode frequency components extracted from the passively mode-locked EDFLs with Si_x_C_1−x_ saturable absorbers. The repetition rate of the mode-locked pulses remains as 29.89 MHz. The upper row of Fig. 6e depicts those component spectra obtained at threshold mode-locking condition of the EDFLs. As usual, the fundamental mode spectrum of the EDFL with Si-rich Si_x_C_1−x_ cannot be observed as which is not mode-locked. When varying the Si_x_C_1−x_ saturable absorber from the stoichiometric to C-rich condition, the spectral peak of fundamental mode increases its carrier-to-noise ratio (CNR) from 13 dB to 42 dB as the C-rich Si_x_C_1−x_ film reveals stronger saturable absorbance and better mode-locking ability than the stoichiometric SiC. By enlarging the pumping power to enable the soliton compression in the EDFL for obtaining sub-400fs pulsewidth, the CNR can be further improved from 27 dB to 56 dB by substituting the stoichiometric SiC with C-rich Si_x_C_1−x_, as shown in the lower row of Fig. 6e. In comparison with the incomplete mode-locking at threshold pumping condition, the soliton pulse exhibits enlarged CNR because of the improved phase coherence among all longitudinal modes contributed to the soliton mode-locking.

**Figure 7:**
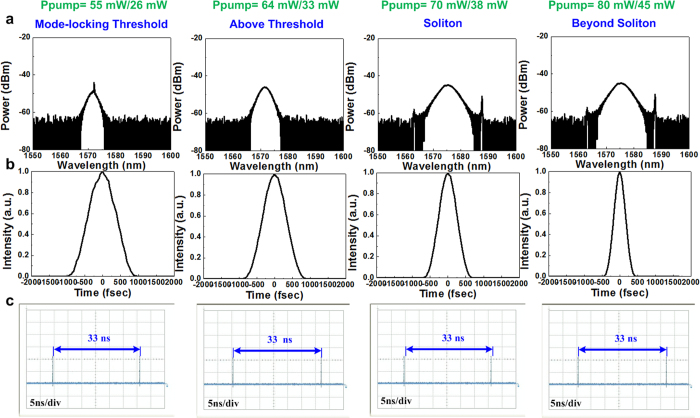
Performance of the EDFL with a C-rich Si_x_C_1−x_ saturable absorber at various pumping operations. (**a**) Optical spectra, (**b**) temporal pulse, and (**c**) pulse train of the passively mode-locked EDFL with a C-rich Si_x_C_1−x_ saturable absorber at different pumping operations.

In more detail, the Fig. 7 illustrates the spectral pulse shapes and pulse trains of the C-rich Si_x_C_1−x_ mode-locked EDFL at various effective gains. These pulse shapes and trains are compared with those of the passively mode-locked EDFL without a saturable absorber. In principle, the C-rich Si_x_C_1−x_ film induces an additional linear loss in the EDFL intracavity, which must be compensated by increasing the pumping power to conduct comparisons at a constant gain. The mode-locked EDFL with a C-rich Si_x_C_1−x_ film effectively suppresses its pulsewidth from 570 to 421 fs by promoting the fundamental mode-locked pulse into a soliton and increasing the forward/backward pumping power from 55 mW/26 mW to 80 mW/45 mW (Fig. 7b). The derived soliton pulse is shorter than that obtained without a saturable absorber because the initial pulse obtained at the SAM stage has already been shortened. The corresponding spectral linewidth (Fig. 7a) is broadened from 4.27 to 6.13 nm, reducing the TBP from 0.327 to 0.316. Moreover, the pulse trains of the mode-locked EDFL with a C-rich Si_x_C_1−x_ saturable absorber shows a clear trace with an identical repetition rate of 30 MHz, and they are not split into multiple soliton bunches (even at extremely high pumping powers) after entering the soliton regime (Fig. 7c).

**Figure 8:**
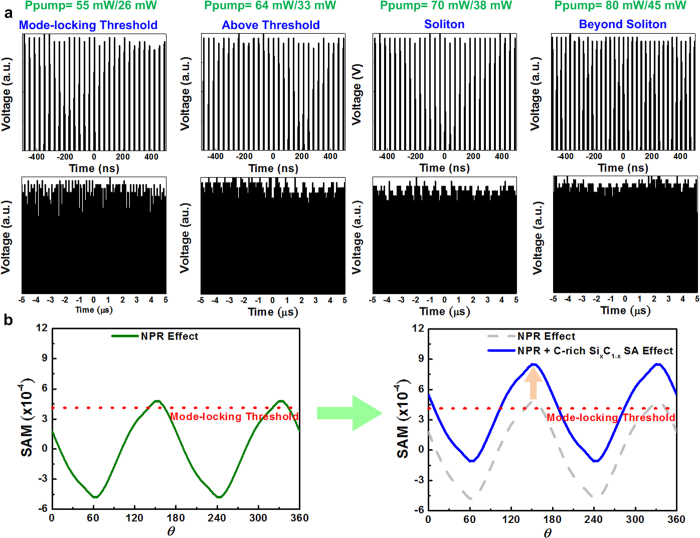
Stability of the EDFL with a C-rich Si_x_C_1−x_ saturable absorber at different pumping operations. (**a**) Stability of the passively mode-locked EDFL with a C-rich Si_x_C_1−x_ saturable absorber at different time scales. (**b**) The SAM coefficient of the EDFL without saturable absorber and with saturable absorber.

Apparently, the C-rich Si_x_C_1−x_ saturable absorber effectively facilitates stable mode locking because it increases the SAM depth and prevents the occurrence of intensity and phase noise in the EDFL pulse to set a higher criterion for passive mode locking. The intensity stability of the passively mode-locked EDFL with a C-rich Si_x_C_1−x_ saturable absorber is examined and compared with that of the EDFL without a saturable absorber (Fig. 8). The CAJ of the EDFL is improved from 1.40% to 0.8% when the forward/backward pumping power is increased from 55 mW/26 mW to 80 mW/45 mW. The CAJ of the EDFL containing a C-rich Si_x_C_1−x_ saturable absorber is lower than that of the EDFL without a saturable absorber because the C-rich Si_x_C_1−x_ saturable absorber induces an additional SAM effect to increase the modulation depth, increasing the stability of the pulse. Regarding the EDFL without a saturable absorber, the pulse became unstable at extremely high pumping powers greater than the fundamental soliton regime because the pulse starts to split into tightly bound soliton pulses. However, the pulse of the EDFL with a C-rich Si_x_C_1−x_ saturable absorber cannot be split into tightly bound soliton pulses. Therefore, the pulse train of the EDFL with a C-rich Si_x_C_1−x_ saturable absorber is more stable at extremely high pumping powers.

### Mechanisms related to the pulse compressing and Kelly sideband frequency shift

Some specific mechanisms related to the pulse compression in the passively mode-locked EDFL are also discussed. Even without using a saturable absorber, but with intracavity polarization controllers and polarizer, the NPR mechanism can be initiated to achieve mode-locking in the EDFL. However, the residual NPR mode-locking is relatively sensitive to the polarization change induced by environment, as which can only be maintained with a small tolerance on the oriented polarization angle. Such a small tolerance contributes to the instability of the passively mode-locked EDFL with its intracavity polarization fluctuated under environmental variations. To evaluate the effect of saturable absorber on releasing the polarization dependence in current EDFL system, the SAM coefficients of the EDFL without and with satiable absorber are simulated and shown in Fig. 8b. It shows that the NPR mode-locking can only be operated within a very narrow range of polarization angle in the EDFL cavity, as the polarization-angle dependent SAM coefficient contributed by the NPR effect reveals a small tolerance to exceed the mode-locking threshold (see Fig. 8b). By adding the C-rich Si_x_C_1−x_ film based saturable absorber into the EDFL cavity, it provides an additional SAM coefficient to extend the polarization angle tolerance for stably mode-locking. In other words, the C-rich Si_x_C_1−x_ saturable absorber offsets the SAM coefficient to allow a larger tolerance on the oriental angle of polarization for stable mode-locking. Moreover, the highly pumped EDFL easily generates the tightly-bunched soliton pulses from the solely NPR mode-locked EDFL without satiable absorber.

In principle, the introduction of spectral filtering and intra-cavity dispersion can affect the pulse duration^42^,^43^. Liu *et al.* have changed the bandwidth of fiber Bragger Grating (FBG) to detune the pulse duration^42^, and the pulsewidth is shortened when increasing the FBG bandwidth. Han *et al.* proposed that the intracavity dispersion can also affect the pulse duration^43^, as analyzed by simultaneously seeding picosecond and femtosecond soliton pulses to observe pulsewidth variation^43^. Indeed, either the detuning of SPM and GDD can contribute to the variation of group delay dispersion on pulse duration at pulse compression stage, which is different mechanism when comparing the SAM effect dominated at initial stage. However, the EDFL has to be maintained at a slightly negative GDD condition to approach the shortest pulsewidth under the desired SPM condition. Therefore, the cavity length cannot be arbitrary detuned so as to ensure the shortest soliton generation at the SPM stage. Besides, the Si_x_C_1−x_ film only induces an extremely small GDD of 2.9 × 10^−8^ ps^2^ when comparing with that of the whole EDFL (−0.16 ps^2^). Under such arrangement in our case, the variation on the pulse duration is mainly dependent on the SAM by Si_x_C_1−x_ films with different C/Si composition ratios. In addition, the increasing GDD and SPM can effectively suppress the soliton pulse after initiating the EDFL pulse by SAM effect^44^. When enlarging the SPM coefficient, the pulsewidth can be effectively compressed. In addition, the enhanced GDD by increasing the SMF length to −0.01 ps^2^ can achieve the optimized soliton pulse with different SPM coefficient.

**Figure 9:**
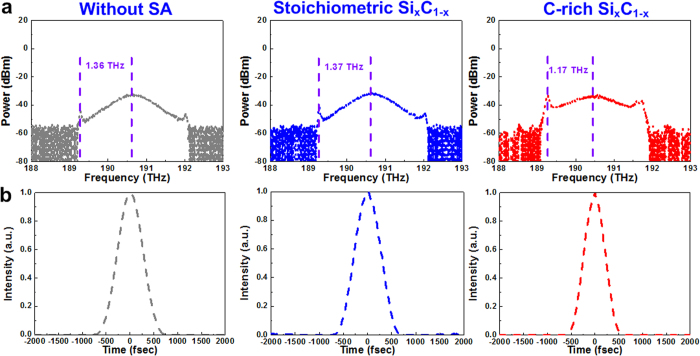
The optical spectra (in frequency domain) and autocorrelation traces obtained from the same EDFL system without and with Si_x_C_1−x_ saturable absorbers. (**a**) Frequencies of Kelly sidebands obtained from the same EDFL system without saturable absorber (left), with the stoichiometric SiC (middle) saturable absorber, and with a C-rich Si_x_C_1−x_ (right) saturable absorber. (**b**) Autocorrelation traces of the same EDFL system without saturable absorber (left), with the stoichiometric SiC (middle) saturable absorber, and with a C-rich Si_x_C_1−x_ (right) saturable absorber.

Figure 9 demonstrates the optical spectra (in frequency domain) and autocorrelation traces of the passively mode-locked EDFLs with Si_x_C_1−x_ saturable absorbers. A Kelly sideband peak with a frequency spacing of 1.36 THz can be observed when only the weak-NPR mode-locking dominates the passive mode-locked EDFL without a saturable absorber. When the stoichiometric Si_x_C_1−x_ saturable absorber is introduced into the EDFL system with a maintained cavity gain (by slightly increasing the pumping power to compensate for the linear loss caused by the introduction of the saturable absorber film), the Kelly sideband frequency spacing of the passively mode-locked EDFL can be acquired as 1.37 THz, which is similar to the spacing shift without saturable absorber. It is because the pulsewidth of the passively EDFL with the stoichiometric SiC is the same as 400 fs. With the C-rich Si_x_C_1−x_ saturable absorber, the Kelly sideband frequency spacing of the passively mode-locked EDFL reduces to 1.17 THz with a shortened pulsewidth of 350 fs.

The frequency spacing of N^th^-order Kelly sideband is related to the effective cavity dispersion and EDFL pulsewiwdth *τ*, as expressed below^45^,


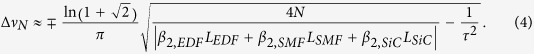


Although non-stoichiometric Si_x_C_1−x_ possesses higher dispersion coefficient *β*_*2,SiC*_ of 145 ps^2^/km than those of SMF (*β*_*2,SMF*_) and EDF (*β*_*2,EDF*_), however, the thickness of Si_x_C_1−x_ film *L*_*SiC*_ is 200 nm, which makes the product of *β*_*2,SiC*_*L*_*SiC*_ (2.9 × 10^−8^ ps^2^) too small to change the GDD (−0.16 ps^2^) dominated by SMF and EDF (*β*_*2,SMF*_*L*_*SMF*_ *+*  *β*_*2,EDF*_*L*_*EDF*_). Therefore, only the variation of pulsewidth can detune the frequency spacing of Kelly sideband. The narrowed EDFL pulsewidth can reduce the frequency spacing of Kelly sideband.

## Discussion

The study presents a novel demonstration of using a PECVD-grown Si_x_C_1−x_ saturable absorber for implementing passive mode-locked EDFLs. The C/Si composition ratio of the Si_x_C_1−x_ films increases from 0.51 to 1.83 when the [CH_4_]/[CH_4_ + SiH_4_] fluence ratio is increased from 70% to 92%; this increase in the fluence ratio corresponds to a drop in the fraction index *x* of the Si_x_C_1−x_ films from 0.66 to 0.33. The Raman scattering signal at 970 cm^−1^ attributed to the LO mode of the Si-C bond is markedly enhanced when the C/Si composition ratio is increased. Furthermore, the Si-related Raman scattering signal at 520 cm^−1^ is attenuated when the C/Si composition ratio is increased from 0.51 to 1.83; this is because a higher quantity of carbon atoms are incorporated into the Si_x_C_1−x_ film during the growing process under CH_4_-rich conditions. Specifically, the sign of the nonlinear absorption coefficient of the Si_x_C_1−x_ film changes from positive to negative as the Si_x_C_1−x_ composition is transformed from Si-rich to C-rich conditions, indicating a variation in the absorption mechanism from a two-photon absorption to nonlinear saturable absorption.

The nonlinear absorption coefficient varies from 4.6 × 10^−6^ to −3.9 × 10^−6^ cm/W as the Si_x_C_1−x_ is transformed from a Si-rich matrix to a C-rich matrix. When the Si-rich Si_x_C_1−x_ film is used as the saturable absorber, the EDFL cannot be mode locked at the same effective gain because the Si-rich Si_x_C_1−x_ film causes higher intracavity linear losses. The EDFL containing the stoichiometric Si_x_C_1−x_ saturable absorber showes an autocorrelated pulsewidth and a spectral linewidth of 492 fs and 5.02 nm, respectively, corresponding to a TBP of 0.321, which was similar to the TBP of the passively mode-locked EDFL without a saturable absorber. This similarity is attributable to the dominance of the weak NPR induced SAM effect. This dominance is also confirmed by the Z-scan and nonlinear transmittance analyses. However, the C-rich Si_x_C_1−x_ film exhibits saturable absorbance along with linear and nonlinear absorbance and the saturation intensity of 0.172, 0.04, and 181 MW/cm^2^, respectively, which is mainly attributed to the sp^2^-orbital C-C bonds. Furthermore, the C-rich Si_x_C_1−x_ saturable absorber can appreciably shorten the pulsewidth to 470 fs, with a corresponding spectral linewidth of 5.13 nm and a TBP of 0.319. This is because the buried sp^2^-orbital C-C bonds enhances the saturable absorption, which dominate the SAM process for mode locking the EDFL. The CAJ of the EDFL with a C-rich saturable absorber is improved from 1.40% to 0.8% by increasing the forward/backward pumping power from 55 mW/26 mW to 80 mW/45 mW because the saturable-absorption-induced SAM process increases the stability of the pulse. When the stoichiometric Si_x_C_1−x_ saturable absorber is inserted into the EDFL system and the intracavity gain is maintained unchanged, the Kelly sideband frequency spacing of the EDFL passively mode locked by a stoichiometric SiC saturable absorber showes a same trend as 1.37 THz. When replacing the stoichiometric SiC saturable absorber by the C-rich Si_x_C_1−x_, the Kelly sideband frequency spacing is decreased to 1.17 THz. This observation is unique and shows that direct evidence of the modification of the EDFL pulsewidth can be easily obtained by monitoring the Kelly sideband shift of the mode-locked EDFL.

Although the SAM coefficient and modulation depth of Si_x_C_1−x_ are lower than those of the graphene, the Si_x_C_1−x_ film still exhibits some advantages as compared to graphene as shown in Table 1. The graphene sample can only be deposited upon specific metal substrates such as copper and nickel. In contrast, the Si_x_C_1−x_ film can be easily deposited on glass, quartz, SiO_2_/Si, Si substrates, etc. Therefore, the Si_x_C_1−x_ film has larger tolerance on substrates selection. In addition, the graphene must be fabricated thinner to enhance its saturated absorption, which increases the difficulty when exfoliating, transferring, or imprinting onto the desired sub substrate. In comparison, the nonlinear loss of the Si_x_C_1−x_ is tunable by changing the C/Si composition ratio, as the saturable absorption enhances when varying Si_x_C_1−x_ film from Si-rich to C-rich condition. This allows the Si_x_C_1−x_ film to be sufficiently thickened for enhancing its robustness during substrate transfer.

## Methods

### Fabrication of the Si_x_C_1−x_ saturable absorber

In the experiment, a 200-nm-thick Si_x_C_1−x_ film was deposited on a Si substrate coated with 3-μm-thick thermal SiO_2_ by using a hydrogen-free low-temperature PECVD system with a gaseous mixture of Ar-diluted silane (Ar-diluted SiH_4_) and methane (CH_4_). The chamber pressure and RF plasma power were controlled at 0.3 Torr and 100 W, respectively. The Ar-diluted SiH_4_ fluence was fixed at 150 sccm, and the fluence ratio, defined as R = [CH_4_]/([CH_4_] + [SiH_4_]), was detuned to 70%, 90%, and 92% for synthesizing the Si-rich, stoichiometric, and C-rich Si_x_C_1−x_ films. The substrate temperature was maintained constant at 500 °C during the synthesis. The C/Si composition ratio of the nonstoichiometric Si_x_C_1−x_ films was determined using XPS with an Mg K_α_-line radiation of 1256.3 eV. The Raman scattering analysis was conducted using a Nd:YAG laser at 532 nm to observe the structural bonds and the crystallization of the Si_x_C_1−x_ films with various C/Si composition ratios.

The nonlinear refractive index of the Si_x_C_1−x_ film was concurrently determined using the Z-scan analysis under femtosecond Ti:sapphire laser (Spectra-Physics, Model Mai Tai VF-TLS) excitation at 800 nm with a pulse width and repetition rate of 80 fs and 80 MHz, respectively. A double-convex lens was used to focus the beam on a spot measuring 20 μm, and the Si_x_C_1−x_ sample was moved along the *z*-axis using a motorized translational stage. The measured peak intensity of the Ti:sapphire laser at the focal point was 1.12 GW/cm^2^. The laser pulse train was modulated using a mechanical chopper (Stanford, SR830) operated at a frequency of 1 kHz, and it was split into a reference beam collected by a photodetector for noise reduction and a pump beam used to measure the position (intensity)-dependent nonlinear transmittance of the Si_x_C_1−x_ films. In the open-aperture Z-scan analysis, the far-field transmitted light was passed through an aperture, and the beam intensity was recorded using a balanced photodetector (New Focus, Model 2307). By contrast, in the closed-aperture Z-scan analysis, only the on-axis divergent beam and diffracted beam were collected using a small aperture collimated with the *z*-axis.

### Architecture of the mode-locked EDFL

**Figure 10:**
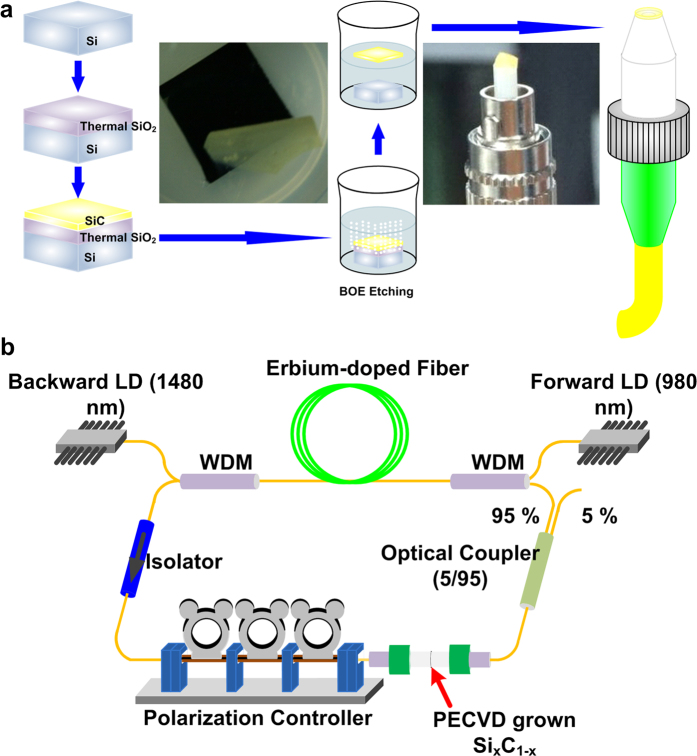
Fabrication of the Si_x_C_1−x_ film and the configuration of the EDFL system. (**a**) Fabrication process for obtaining a PECVD-grown Si_x_C_1−x_ film. The two photographs show the PECVD-grown Si_x_C_1−x_ film being lifted off under a buffer oxide etching solution and a PECVD-grown Si_x_C_1−x_ film on a SMF. (**b**) Configuration of the passively mode-locked EDFL with a Si_x_C_1−x_ saturable absorber.

To lift off a Si_x_C_1−x_ film from a SiO_2_/Si wafer, the entire Si_x_C_1−x_/SiO_2_/Si sample was immersed in a buffer oxide etching solution to remove the SiO_2_ buffer (Fig. 10a). The Si_x_C_1−x_ film was clearly separated from the SiO_2_/Si wafers. Subsequently, the Si_x_C_1−x_ film was picked using a tweezer and directly covered on the end-face of an SMF patchcord. Figure 10b illustrates the configuration of the passively mode-locked EDFL system with the PECVD-grown Si_x_C_1−x_ saturable absorber adhered between two connector end-faces. A 2-m-long EDF (Liekki Er80-8/125) was employed as the gain medium, and its dispersion coefficient (*β*_*2, EDF*_) was −20 ps^2^/km. The ring-type EDFL system was comprised by two high-power pumping laser diodes with central wavelengths of 980 (forward pumping) and 1480 nm (backward pumping); 980 nm/C-band and 1480 nm/C-band wavelength division multiplex couplers were also employed for coupling the pumping and circulated lights. A nonpolarized isolator was used to determine the direction of light propagated in the EDFL, and the polarization of the circulated light was controlled by a polarization controller located before the Si_x_C_1−x_ saturable absorber. A 1 × 2 optical coupler was used to provide 95% feedback and 5% output. The total length of the SMF with *β*_*2,SMF*_ = −21 ps^2^/km was 4.7 m in the EDFL cavity, thus providing a total GDD of −0.16 ps^2^ in the EDFL cavity. The temporal shape and optical spectrum of the passively mode-locked EDFL pulse were measured using an autocorrelator (Femtochrome, FR-103XL) and an optical spectrum analyzer (Ando, AQ6317B), respectively.

## Additional Information

**How to cite this article**: Cheng, C.-H. *et al.* Can silicon carbide serve as saturable absorber for passively mode-locked fiber lasers? *Sci. Rep.* **5**, 16463; doi: 10.1038/srep16463 (2015).

## Figures and Tables

**Table 1:** The characteristic parameters of saturable absorbance of the graphene and C-rich Si_x_C_1−x_.

Material	T	Psat	α_lin_	α_non_	M_D_	γ
Graphene	91%	0.8	0.085	0.05	0.26	7.6 × 10^−4^
C-rich Si_x_C_1−x_	81%	3.81	0.172	0.045	0.14	3.9 × 10^−4^

## References

[b1] Kieu K., Mehravar S., Gowda R., Norwood R. A. & Peyghambarian N. Label-free multi-photon imaging using a compact femtosecond fiber laser mode-locked by carbon nanotube saturable absorber. Biomed. Opt. Express 4, 2187–2195 (2013). 24156074 10.1364/BOE.4.002187 PMC3799676

[b2] Gattass R. R. & Mazur E. Femtosecond laser micromachining in transparent materials. Nat. Photonics 2, 219–225 (2008).

[b3] Quinlan F. *et al.* Ultralow phase noise microwave generation with an Er:fiber-based optical frequency divider. Opt. Lett. 36, 3260–3262 (2011). 21847227 10.1364/OL.36.003260

[b4] Sato K., Kotaka I., Kondo Y. & Yamamoto M. Active mode locking at 50 GHz repetition frequency by half-frequency modulation of monolithic semiconductor lasers integrated with electroabsorption modulators. Appl. Phys. Lett. 69, 2626–2628 (1996).

[b5] Yoshida E. & Nakazawa M. 80 ~ 200 GHz erbium doped fibre laser using a rational harmonic mode-locking technique. Electron. Lett. 32, 1370–1372 (1996).

[b6] Brabec T., Spielmann C., Curley P. F. & Krausz F. Kerr lens mode locking. Opt. Lett. 17, 1292–1294 (1992). 19798161 10.1364/ol.17.001292

[b7] Zhang Z. *et al.* Self-starting mode-locked femtosecond forsterite laser with a semiconductor saturable-absorber mirror. Opt. Lett. 22, 1006–1008 (1997). 18185734 10.1364/ol.22.001006

[b8] Yamashita S. *et al.* 5-GHz pulsed fiber Fabry-Perot laser mode-locked using carbon nanotubes. IEEE Photonic. Tech. Lett. 17, 750–752 (2005).

[b9] Bao Q. *et al.* Monolayer Graphene as a Saturable Absorber in a Mode-Locked Laser. Nano Res. 4, 297–307 (2011).

[b10] Lee J., Koo J., Jhon Y. M. & Lee J. H. A femtosecond pulse erbium fiber laser incorporating a saturable absorber based on bulk-structured Bi_2_Te_3_ topological insulator. Opt. Express 22, 6165–6173 (2014). 24663950 10.1364/OE.22.006165

[b11] Keller U. *et al.* Semiconductor Saturable Absorber Mirrors (SESAM’s) for Femtosecond to Nanosecond Pulse Generation in Solid-State Lasers. IEEE J. Sel. Top. Quantum Electron. 2, 435–453 (1996).

[b12] Jung I. D. *et al.* Semiconductor saturable absorber mirrors supporting sub-10-fs pulses. Appl. Phys. B-Lasers O. 65, 137–150 (1997).

[b13] Set S. Y., Yaguchi H., Tanaka Y. & Jablonski M. Laser mode locking using a saturable absorber incorporating carbon nanotubes. J. Lightwave Technol. 22, 51–56 (2004).

[b14] Kataura H. *et al.* Optical properties of single-wall carbon nanotubes. Synthetic Met. 103, 2555–2558 (1999).

[b15] Weisman R. B. & Bachilo S. M. Dependence of optical transition energies on structure for single-walled carbon nanotubes in aqueous suspension: An empirical kataura plot. Nano lett. 3, 1235–1238 (2003).

[b16] Sun Z., Hasan T. & Ferrari A. C. Ultrafast lasers mode-locked by nanotubes and grapheme. Physica E 44, 1082–1091 (2012).

[b17] Bao Q. *et al.* Atomic-layer graphene as a saturable absorber for ultrafast pulsed lasers. Adv. Funct. Mater. 19, 3077–3083 (2009).

[b18] Lin Y.-H., Yang C.-Y., Liou J. H., Yu C. P. & Lin G.-R. Using graphene nano-particle embedded in photonic crystal fiber for evanescent wave mode-locking of fiber laser. Opt. Express 21, 16763–16776 (2013). 23938528 10.1364/OE.21.016763

[b19] Xu J. L. *et al.* Graphene saturable absorber mirror for ultra-fast-pulse solid-state laser. Opt. Lett. 36, 1948–1950 (2011). 21593945 10.1364/OL.36.001948

[b20] Lin Y.-H. & Lin G.-R. Free-standing nano-scale graphite saturable absorber for passively mode-locked erbium doped fiber ring laser. Laser Phys. Lett. 9, 398–404 (2012).

[b21] Lin Y.-H. & Lin G.-R. Kelly sideband variation and self four-wave-mixing in femtosecond fiber soliton laser mode-locked by multiple exfoliated graphite nano-particles. Laser Phys. Lett. 10, 045109 (2013).

[b22] Xu J., Liu J., Wu S., Yang Q.-H. & Wang P. Graphene oxide mode-locked femtosecond erbium-doped fiber lasers. Opt. Express 20, 15474–15480 (2012). 22772242 10.1364/OE.20.015474

[b23] Sobon G. *et al.* Graphene Oxide vs. Reduced Graphene Oxide as saturable absorbers for Er-doped passively mode-locked fiber laser. Opt. Express 20, 19463–19473 (2012). 23038589 10.1364/OE.20.019463

[b24] Lin Y.-H., Chi Y.-C. & Lin G.-R. Nanoscale charcoal powder induced saturable absorption and mode-locking of a low-gain erbium-doped fiber-ring laser. Laser Phys. Lett. 10, 055105 (2013).

[b25] Lin Y. H., Lo J. Y., Tseng W. H., Wu C.-I. & Lin G.-R. Self-amplitude and self-phase modulation of the charcoal mode-locked erbium-doped fiber lasers. Opt. Express 21, 25184–25196 (2013). 24150360 10.1364/OE.21.025184

[b26] Zhao C. J. *et al.* Wavelength-tunable picosecond soliton fiber laser with Topological Insulator: Bi_2_Se_3_ as a mode locker. Opt. Express 20, 27888–27895 (2012). 23262733 10.1364/OE.20.027888

[b27] Zhao C. J. *et al.* Ultra-short pulse generation by a topological insulator based saturable absorber. Appl. Phys. Lett. 101, 211106 (2012).

[b28] Currie M. *et al.* Quantifying pulsed laser induced damage to graphene. Appl. Phys. Lett. 99, 211909 (2011).

[b29] Lefèvre J. Costantini J.-M., Esnouf S. & Petite G. Thermal stability of irradiation-induced point defects in cubic silicon carbide. J. Appl. Phys. 106, 083509 (2009).

[b30] DesAutels G. L. *et al.* Femtosecond laser damage threshold and nonlinear characterization in bulk transparent SiC materials. J. Opt. Soc. Am. B 25, 60–66 (2008).

[b31] Huran J., Hrubcin L., Kobzev A. P. & Liday J. Properties of amorphous silicon carbide films prepared by PECVD. Vaccum 47, 1223–1225 (1996).

[b32] Demichelisa F. *et al.* The influence of hydrogen dilution on the optoelectronic and structural properties of hydrogenated amorphous silicon carbide films. Philos. Mag. B 69, 377–386 (1994).

[b33] Ding J.-J. *et al.* Nonlinear optical properties and ultrafast dynamics of undoped and doped bulk SiC. Chin. Phys. Lett. 27, 124202 (2010).

[b34] Cheng C.-H. *et al.* Si-rich Si_x_C_1−x_ light-emitting diodes with buried Si quantum dots. IEEE Photonics J. 4, 1762–1775 (2012).

[b35] Feng Z. C., Mascarenhas A. J., Choyke W. J. & Powell J. A. Raman scattering studies for chemical vapor deposited 3C-SiC films on (100) Si. J. Appl. Phys. 64, 3176–3186 (1988).

[b36] Ferrari A. C. Raman Spectroscopy of Graphene and Graphite: Disorder, Electron-Phonon Coupling, Doping and Nonadiabatic Effect. Solid State Commun. 143, 47–57 (2007).

[b37] Prawer S. & Nemanich R. J. Raman spectroscopy of diamond and doped diamond. Phil. Trans. R. Soc. Lond. A 362, 2537–2565 (2004). 10.1098/rsta.2004.1451 15482990

[b38] Sheik-Bahae M., Said A. A., Wei T.-H., Hagan D. J. & Stryland E. W. V. Sensitive measurement of optical nonlinearities using a single beam. IEEE J. Quantum Electron. 26, 760–769 (1990).

[b39] Boyd R. W. The Nonlinear Optical Susceptibility. In Nonlinear Optics Ch. 1, pp. 33, Elsevier (2008).

[b40] Cowana B. M. Optical damage threshold of silicon for ultrafast infrared pulses. Proc. SPIE 6720, 67201M (2007).

[b41] Reitze D. H., Wang X., Ahn H. & Downer M. C. Femtosecond laser melting of graphite. Phys. Rev. B 40, 11986–11989 (1989). 10.1103/physrevb.40.11986 9991819

[b42] Liu X. M. & Cui Y. D. Flexible pulse-controlled fiber laser. Sci. Rep. 5, 9399 (2015). 25801546 10.1038/srep09399 PMC4371082

[b43] Han D. D. *et al.* Simultaneous picosecond and femtosecond solitons delivered from a nanotube-mode-locked all-fiber laser. Opt. Lett. 39, 1565–1668 (2014). 24690839 10.1364/OL.39.001565

[b44] Yang C.-Y. *et al.* Pulse-width saturation and Kelly-sideband shift in a graphene-nanosheet mode-locked fiber laser with weak negative dispersion. Phys. Rev. Appl. 3, 044016 (2015).

[b45] Kelly S. M. J. Characteristic sideband instability of periodically amplified average soliton. Electron. Lett. 28, 806–807 (1992).

