# Corrigendum: Can silicon carbide serve as a saturable absorber for passive mode-locked fiber lasers?

**DOI:** 10.1038/srep21056

**Published:** 2016-03-10

**Authors:** Chih-Hsien Cheng, Yung-Hsiang Lin, Ting-Hui Chen, Hsiang-Yu Chen, Yu-Chieh Chi, Chao-Kuei Lee, Chih-I Wu, Gong-Ru Lin

Scientific Reports 5: Article number: 16463; 10.1038/srep16463 published online: 11122015; updated: 03102016

The original version of this Article contained typographical errors in the spelling of the authors Chao-Kuei Lee and Chih-I Wu which were incorrectly given as Chao-Kuei Leeb and Chih-I Wua respectively.

In addition, there were errors in the Acknowledgements section.

“The authors thank the Ministry of Science and Technology, Taiwan, R.O.C., and the Excellent Research Projects of National Taiwan University, Taiwan, for financially supporting this research under grants NSC 101-2221-E-002-071-MY3, and MOST 103-2221-E002-042-MY3, 104-2221-E-002 -117 -MY3, 103R89081 and 103R89083.”

now reads:

“The authors thank the Ministry of Science and Technology, Taiwan, R.O.C., and Excellent Research Projects of the National Taiwan University, Taiwan, R.O.C., for financially supporting this research under grants MOST 103-2221-E002-042-MY3, MOST- 104-2221-E-002 -117 -MY3, NTU-ERP-105R89081 and NTU-ERP-105R89083.”

These errors have now been corrected in the PDF and HTML versions of the Article.

